# Unraveling the molecular principles by which ceramides commit cells to death

**DOI:** 10.15698/cst2019.08.196

**Published:** 2019-07-16

**Authors:** Shashank Dadsena, Dina G. Hassan, Joost C.M. Holthuis

**Affiliations:** 1 Molecular Cell Biology Division, Department of Biology/Chemistry and Center for Cellular Nanoanalytics, Osnabrück University, 49076 Osnabrück, Germany.

**Keywords:** photo-activatable lipids, voltage-dependent anion channel, mitochondrial apoptosis, tumor suppressor lipid, hexokinase, ceramide transfer protein

## Abstract

Ceramides are central intermediates of sphingolipid metabolism that can activate a variety of tumor suppressive cellular programs, including cell cycle arrest, senescence and apoptosis. Indeed, perturbations in ceramide generation and turnover are frequently linked to cancer cell survival and resistance to chemotherapy. Consequently, the potential of ceramide-based therapeutics in the treatment of cancer has become a major focus of interest. A growing body of evidence indicates that ceramides can act directly on mitochondria to trigger apoptotic cell death. However, molecular details of the underlying mechanism are scarce. In our recent study (Dadsena S *et al.*, 2019, Nat Commun 10:1832), we used a photoactivatable ceramide probe combined with computer simulations and functional studies to identify the voltage-dependent anion channel VDAC2 as a critical effector of ceramide-induced mitochondrial apoptosis. Collectively, our findings provide a novel molecular framework for how ceramides execute their widely acclaimed anti-neoplastic activities.

## EMERGENCE OF CERAMIDES AS TUMOR SUPPRESSOR LIPIDS

Sphingolipids are unusually versatile membrane components that participate in mechanical stability, signaling, and molecular sorting. As the path to their production is paved with toxic intermediates, cells must monitor sphingolipid biosynthesis closely to avoid jeopardizing their viability. Notably, ceramides, the direct precursors of all complex sphingolipids, are potent mediators of cell cycle arrest, stress signaling and apoptosis. Ceramide engagement in apoptosis is clinically relevant. Many anticancer regimens, including ionizing radiation and chemotherapeutic agents, have been shown to trigger ceramide-mediated cell death. While these findings raised considerable interest in targeting ceramide-metabolic enzymes for cancer therapy, mechanistic insights into how ceramides exert their tumor suppressor activities are limited. Converging lines of evidence indicate that ceramides can act directly on mitochondria to promote permeabilization of the outer mitochondrial membrane (OMM) for cytochrome *c*, a major ingredient of a cocktail of pro-apoptotic proteins that in healthy cells is sequestered by mitochondria. The release of cytochrome *c* into the cytosol is a point of no return in the suicide program, initiating activation of caspases that execute an ordered self-destruction of the cell. Consequently, how a rise in mitochondrial ceramides triggers the release of cytochrome *c* is a hotly debated issue. One model postulates that ceramides can self-assemble into channels in the OMM that are large enough to enable passage of cytochrome *c.* An alternative view is that ceramides form lipid microdomains into which the pro-apoptotic Bcl2 protein Bax inserts and oligomerizes into a cytochrome *c*-conducting pore. In addition, some experiments with purified mitochondria indicate that ceramides may require metabolic conversion to gain apoptogenic activity.

## LIPID TRANSFER PROTEINS AS TOOLS TO DISSECT THE MECHANISM OF CERAMIDE-MEDIATED CELL DEATH

**Figure 1:**
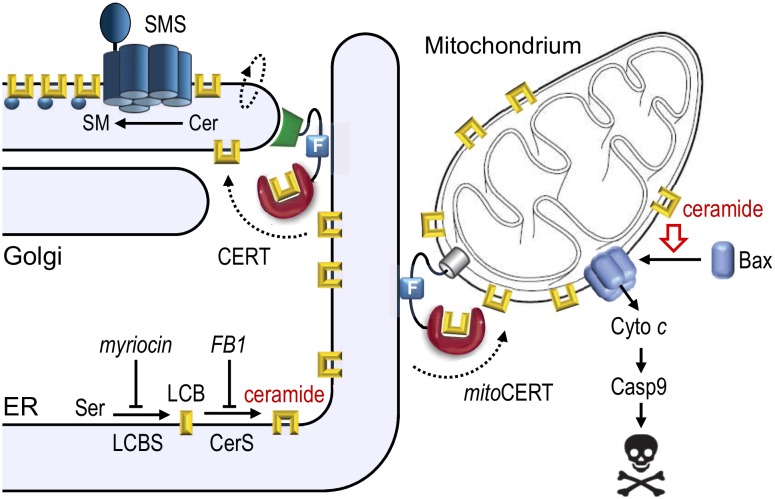
FIGURE 1: Mitochondrial targeting of CERT induces Bax-dependent apoptosis. Ceramides are synthesized through *N*-acylation of long-chain bases (LCB) by ceramide synthases (CerS) on the cytosolic surface of the endoplasmic reticulum (ER). The bulk of newly synthesized ceramides are transferred to the Golgi by the ceramide transfer protein CERT for metabolic conversion into sphingomyelin by a Golgi-resident SM synthase (SMS). Expression of mitoCERT, a CERT variant in which the Golgi-targeting pleckstrin homology domain was replaced by an outer mitochondrial membrane anchor, triggers Bax-dependent apoptosis by diverting the biosynthetic flow of ceramides to mitochondria. Figure adapted from Dadsena *et al.* (2019), Nat Commun 10:1832. doi: 10.1038/s41467-019-09654-4

Most previous studies on ceramide-activated cell death pathways relied on the application of truncated ceramide analogues, isolated mitochondria and/or the treatment of cells with apoptotic stimuli that affect ceramide pools in multiple organelles. To circumvent some of the drawbacks of these approaches, we have begun to exploit lipid transfer proteins as experimental tools. By mediating lipid transport at contact sites between two organelles, these proteins enable lipids to reach their destination independent of vesicular trafficking. Ceramides are synthesized *de novo* on the cytosolic surface of the endoplasmic reticulum (ER). In mammals, the bulk of newly synthesized ceramides is transported to the Golgi by a ceramide transfer protein, CERT, for metabolic conversion into sphingomyelin (SM) by a Golgi-resident SM synthase. Thus, newly synthesized ceramides largely bypass vesicular trafficking to reach the Golgi, where they run in a metabolic trap set by SM synthase. An obvious advantage of this arrangement is that it allows cells to readily clear the ER of a potentially toxic class of metabolic intermediates that are synthesized there. To study the consequences of diverting the biosynthetic flow of ceramides to mitochondria, we designed a CERT variant equipped with an OMM anchor, mitoCERT **(Fig. 1)**. We found that cells expressing mitoCERT import newly-synthesized ceramides into mitochondria and undergo Bax-dependent apoptosis. Apoptosis induction by mitoCERT was blocked by mitochondrial targeting of a bacterial ceramidase, indicating that apoptogenic activity relies on intact ceramides rather than any downstream metabolic intermediate of ceramide turnover. These results confirmed that a rise in mitochondrial ceramides suffices to activate Bax and trigger apoptosis.

## IDENTIFICATION OF VDAC2 AS A DIRECT EFFECTOR OF CERAMIDE-MEDIATED CELL DEATH

The foregoing raised the question whether ceramide-mediated activation of Bax relies on direct and specific interactions between Bax and ceramides or involves other mitochondria-resident proteins. This led us to use a photoactivatable and clickable ceramide probe in a screen for mitochondrial ceramide binding proteins. Photoaffinity labeling experiments with purified mitochondria consistently revealed a 33 kDa protein that was efficiently crosslinked by the ceramide probe. Affinity purification and mass spectrometric analysis revealed that this 33 kDa protein actually corresponds to two proteins, namely the voltage-dependent anion channels VDAC1 and VDAC2. Besides mediating metabolic crosstalk between mitochondria and the cytosol, VDAC2 serves as a mitochondrial platform for Bax translocation whereas VDAC1 oligomerization has been implicated as a critical step in mitochondrial apoptosis. Molecular dynamics simulations revealed that both channels harbor a ceramide binding site buried in the membrane interior on one side of the barrel wall. This site includes a uniquely positioned glutamate (Glu) that faces the bilayer's hydrophobic core and that has a p*K*a valuethat is closely tuned to the physiological pH of the cytosol. In its deprotonated fully charged state, this residue could be seen to mediate direct contact with the ceramide head group. Protonation, substitution or chemical modification of the Glu residue in each case abolished photolabeling of both channels with the ceramide probe. Moreover, photolabeling was progressively reduced when carried out in excess of native ceramides, indicating that the charged membrane-buried Glu is part of an authentic ceramide binding site. To address whether VDACs play a role in ceramide-induced apoptosis, we next determined the impact of VDAC removal on mitoCERT-mediated cell death in human HCT116 colon carcinoma cells. Unlike VDAC1 removal, loss of VDAC2 rendered the cells resistant to mitoCERT-mediated cell death. Importantly, substitution of the membrane-buried Glu in VDAC2 greatly reduced its ability to restore mitoCERT-mediated cell death in VDAC1/2 double knockout cells. Collectively, these findings qualified VDAC2 as a direct and critical effector of ceramide-induced apoptosis.

## A MOLECULAR FRAMEWORK FOR HOW CERAMIDES EXERT THEIR ANTI-NEOPLASTIC ACTIVITIES

**Figure 2:**
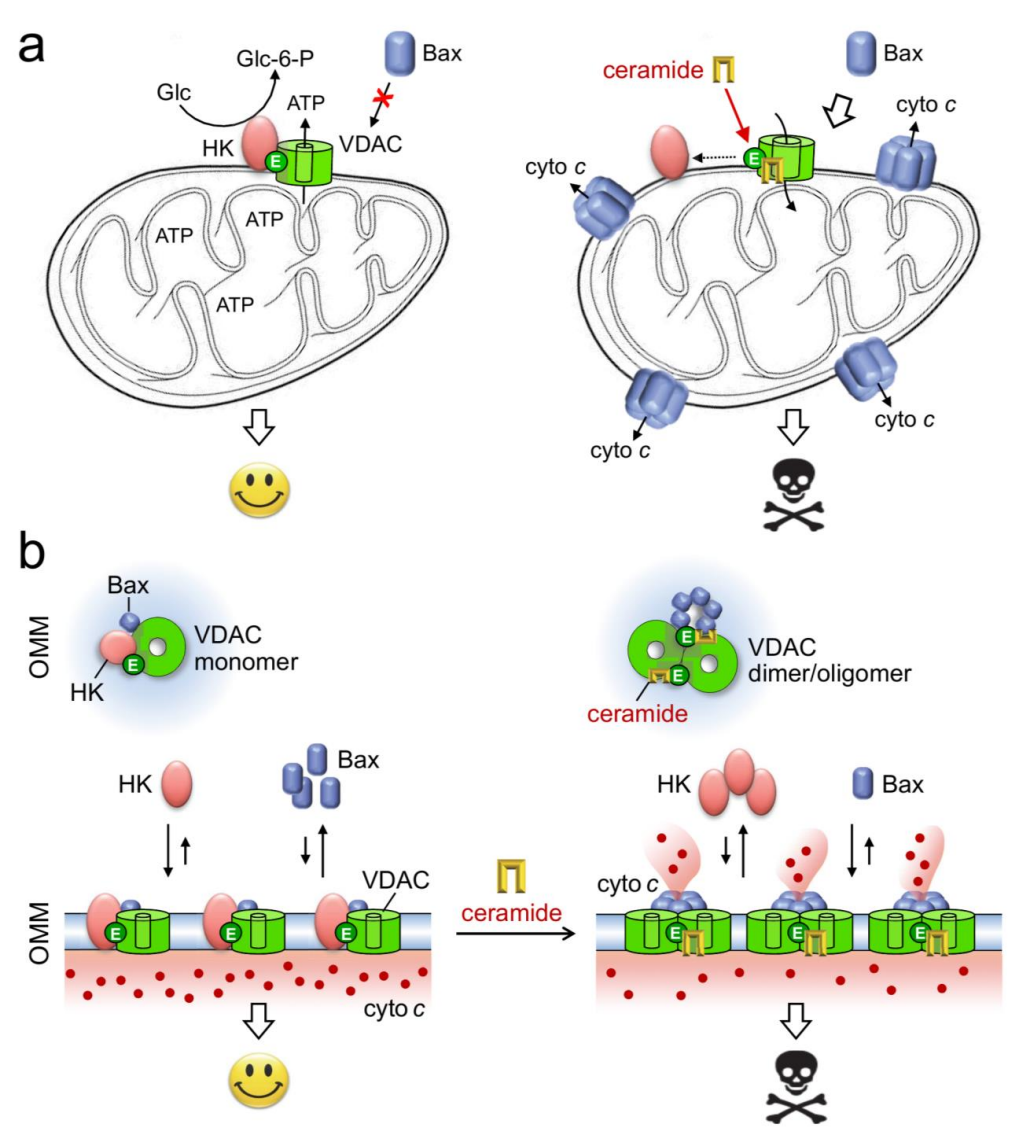
FIGURE 2: Model of how ceramides may exert their tumor suppressor activities. **(A)** Under non-stressed conditions, the bulk of hexokinases (HK) is bound to VDACs. This interaction antagonizes cell death by blocking mitochondrial translocation of Bax and renders cancer cells hyperglycolytic by enhancing the phosphorylation of glucose, the rate-limiting step in glycolysis. As ceramides bind VDACs at a site that overlaps with the binding site for HK, the arrival of these stress mediators in mitochondria may promote dissociation of HK from VDACs. This would revert the hyperglycolytic state of cancer cells as well as sensitize them to Bax-dependent apoptosis. **(B)** Binding of HK and ceramide to VDACs in each case is critically dependent on a Glu residue that faces the hydrophobic membrane interior. We postulate that ceramide binding facilitates VDAC dimerization/oligomerization, a process that hinders the association of HK and helps create mitochondrial platforms for Bax translocation. See text for further details.

Previous work revealed that the charged membrane-buried Glu in VDACs is essential for binding of hexokinase I, which antagonizes cell death through inhibition of Bax-mediated release of cytochrome *c*. Overexpression of hexokinases and their association with VDACs are typical features of hyperglycolytic cancer cells. When bound to VDACs, hexokinases enable an effective coupling between oxidative phosphorylation and glycolysis by capturing ATP released from mitochondria to phosphorylate glucose, the rate-limiting step in the glycolytic cascade. In the clinic, there is great interest in drugs that dissociate hexokinases from VDACs, as such drugs would have a dual anti-neoplastic effect. First, they would facilitate induction of apoptosis. Second, they would revert the hyperglycolytic state that allows tumors to grow under conditions where oxygen is in short supply. Our finding that ceramides bind VDACs at a site that overlaps with the binding site for hexokinases implies that ceramides may exert their tumor suppressor activities in part by influencing the interaction between VDACs and hexokinases **(Fig. 2A)**. How could this work? Our simulations and photoaffinity labeling studies indicate that ceramide binding to VDACs is pH sensitive and controlled by the protonation state of the membrane-buried Glu. Interestingly, acidification has been shown to promote association of two VDAC1 monomers into a dimer, presumably by enabling formation of H-bonds between the Glu and a serine residue at the dimer interface. Under normal stress-free conditions, the membrane-buried Glu is in its deprotonated fully charged state at least a significant amount of time, thus keeping VDACs at the brink of oligomerization. We speculate that binding of ceramides to the negatively charged Glu may lower the threshold for VDAC oligomerization at neutral pH. Such ceramide-driven oligomerization of VDACs may then trigger apoptosis by: i) blocking binding of anti-apoptotic hexokinases; ii) creating a suitable platform for mitochondrial translocation of Bax **(Fig. 2B)**. In sum, our recent study provides a novel molecular framework that will serve as a valuable guide to further unravel how ceramides execute their tumor suppressor activities.

